# Host species and habitat shape fish-associated bacterial communities: phylosymbiosis between fish and their microbiome

**DOI:** 10.1186/s40168-023-01697-6

**Published:** 2023-11-20

**Authors:** Javad Sadeghi, Subba Rao Chaganti, Timothy B. Johnson, Daniel D. Heath

**Affiliations:** 1 Great Lakes Institute for Environmental Research, University of Windsor, Windsor, ON N9B 3P4 Canada; 2 Cooperative Institute for Great Lakes Research, University of Michigan, Ann Arbor, MI USA; 3 Ontario Ministry of Natural Resources and Forestry, Glenora Fisheries Station, Picton, ON Canada; 4 Department of Integrative Biology, University of Windsor, Windsor, ON Canada

**Keywords:** Host-microbe, Microbial communities, Microbial ecology, Microbiome, Phylosymbiosis

## Abstract

**Background:**

While many studies have reported that the structure of the gut and skin microbiota is driven by both species-specific and habitat-specific factors, the relative importance of host-specific versus environmental factors in wild vertebrates remains poorly understood. The aim of this study was to determine the diversity and composition of fish skin, gut, and surrounding water bacterial communities (hereafter referred to as microbiota) and assess the extent to which host habitat and phylogeny predict microbiota similarity. Skin swabs and gut samples from 334 fish belonging to 17 species were sampled in three Laurentian Great Lakes (LGLs) habitats (Detroit River, Lake Erie, Lake Ontario). We also collected and filtered water samples at the time of fish collection. We analyzed bacterial community composition using 16S metabarcoding and tested for community variation.

**Results:**

We found that the water microbiota was distinct from the fish microbiota, although the skin microbiota more closely resembled the water microbiota. We also found that environmental (sample location), habitat, fish diet, and host species factors shape and promote divergence or convergence of the fish microbiota. Since host species significantly affected both gut and skin microbiota (separately from host species effects), we tested for phylosymbiosis using pairwise host species phylogenetic distance versus bacterial community dissimilarity. We found significant phylogenetic effects on bacterial community dissimilarity, consistent with phylosymbiosis for both the fish skin and gut microbiota, perhaps reflecting the longstanding co-evolutionary relationship between the host species and their microbiomes.

**Conclusions:**

Analyzing the gut and skin mucus microbiota across diverse fish species in complex natural ecosystems such as the LGLs provides insights into the potential for habitat and species-specific effects on the microbiome, and ultimately the health, of the host.

Video Abstract

**Supplementary Information:**

The online version contains supplementary material available at 10.1186/s40168-023-01697-6.

## Introduction

Host-associated microbiomes, specifically the bacterial community (hereafter referred to as microbiota) present inside the host as well as on body surfaces, influence a broad range of host immunological, evolutionary, and ecological processes [1, 2]. The term “microbiome” refers to the entire microbial ecosystem, including the microorganisms (prokaryotes and eukaryotes), their genomes, and their surrounding habitats [3], and is hypothesized to have co-evolved with its host [4]. Significant research effort has focused on the importance of exogenous abiotic and biotic factors (e.g., habitat, geography, microbial biodiversity, diet) and endogenous host-related factors (e.g., genetics, physiology, immunity) in driving the composition of the microbiome [5–7]. At the microbial species level, deterministic (endogenous and exogenous) factors are thought to be the dominant forces shaping species composition of microbiota [8–10]. On the other hand, stochastic process-driven microbiome assembly (population growth rates, colonization events, extinction, and speciation) assumes that bacterial species are essentially functionally equivalent (neutral), and community assembly is the result of stochastic dispersal and drift through which organisms are randomly lost and replaced [11, 12]. Recent studies showed that both stochastic and deterministic processes are shaping the microbiome, but still a central question is the extent to which these two processes influence the host, as well as all of their associated microbes [10, 13]. Despite the known effects of habitat and host-specific factors on the microbiome across host taxa, very little is known about the degree of variation in fish-associated microbiota diversity and composition that occurs within and among species in the wild [14]. Moreover, to know how host-associated microbiota may influence host phenotype, and hence contribute to evolutionary processes, quantifying the degree and nature of among-host species microbiome variation and the systematic drivers behind it seems crucial.

We expect that hosts and their microbiomes are linked eco-evolutionarily and the microbiome composition will recapitulate the phylogeny of their host, the basis of phylosymbiosis theory [15]. Essentially, this predicts that hosts that are phylogenetically similar will have microbiomes that are more similar and vice versa [16]. Phylosymbiosis may occur through stochastic and/or deterministic processes [17], mirroring genetic evolution by drift and/or selection. However, patterns of phylosymbiosis are also expected to be affected by exposure to habitat microbiome(s) [18]. Although most studies in which phylosymbiosis has been identified have focused on microbes inhabiting internal organs, such as the gastrointestinal tract [15], recent work suggests that external host microbiomes (e.g., skin) can also exhibit a phylosymbiosis signal [19]. In expanding the range of studied vertebrate microbiomes, questions about the range of environmental, ecological, and evolutionary factors that shape gut and skin microbiomes (or more specifically, bacterial communities), and the functions of those communities, still remained unanswered. For example, similar gut microbiome are found among phylogenetically related mammals but also among unrelated mammal species with similar diets [20–22].

Most host-microbial interaction studies are mainly focused on mammalian species, with fewer studies focused on other species [23]. Teleosts encompass over half of vertebrate diversity [24] and are one of the most successful groups of vertebrates on Earth [25]. Teleosts are represented by more than 32,000 species, originated over 600 million years ago, and exhibit a variety of physiologies, natural/life histories, and ecologies [26]. However, their success and species radiation would not have been possible without the help of their microbiome [27]. Additionally, their long history of co-evolution and symbiotic relationships with microbes (compared to mammals which evolved 160 million years ago [21]) make them good candidates to study host-microbial interactions. However, while most published studies of fish microbiomes include the gut microbiome, few studies have included other key microbial habitats such as skin [28, 29]. Fish skin mucosal immune responses play substantial roles in the course of infection prevention, and healthy skin (mucus) microbial communities are a critical component of that response [30]. In fact, some beneficial bacteria on fish skin can produce antimicrobial compounds which help fish fight pathogenic microorganisms [31, 32]. Moreover, while fish skin is continuously exposed to numerous microorganisms (mainly from water sources), the skin can discriminate between beneficial microbiota and pathogenic microorganisms, although the mechanism is not fully understood [33]. The role of the skin microbiome in host health is under-studied and, importantly for this study, the skin microbiome may thus be under separate selective pressures from the gut microbiome [14].

The Laurentian Great Lakes (LGLs) in North America form the largest freshwater ecosystem on the planet and have provided valuable ecosystem services for humans for centuries [34]. The LGLs and their associated drainages include diverse ecosystems, complex trophic interactions, and mixed watersheds including forested, wetland, agricultural, and urban areas. The LGLs thus represent a powerful natural ecological laboratory to study important questions concerning microbial ecology and host-microbe interactions and co-evolution. While much work has been done on characterizing microbial communities in LGLs themselves, there is a critical need to examine the roles of host species and habitat on the microbiome of fish at the fish community level. The fish and microbial communities of LGLs provide a novel opportunity to study the intra- and interspecific divergence of host-associated intestinal and skin microbiota among the diverse species of the fish communities.

The aim of this study was to characterize fish bacterial communities among a variety of host species across three locations to (i) identify patterns of fish-associated bacterial communities, (ii) quantify the similarity among water bacterial communities and those of the fishes, and (iii) determine the extent to which microbiota among fish follow a pattern of phylosymbiosis. Our first hypothesis is that host-related deterministic processes (host-based selection pressures on bacteria composition) are the main drivers of bacterial community composition variation, and hence most bacterial community composition variation will be found among species. Our second hypothesis is that the water microbiota will be distinct from the host-associated microbiota, and the gut microbiota will also be different from the skin microbiota, but more similar to the water microbiota than the gut. Finally, we predict that the gut microbiota will be more strongly correlated with host phylogeny than the skin microbiota, as the skin microbiota will be affected by the local environmental microbiome more strongly. Characterizing intraspecific variation in fish microbiomes will help managers, particularly in aquaculture settings, effectively manipulate gut and skin microbiome to promote animal health and well-being. On the other hand, such information may provide fish managers tools to assess fish stock status and health. Specifically, skin swab samples could be used as a non-invasive sampling method to assess the health status of the fish, a valuable option for rare and at-risk fish species. More broadly, demonstrating the possible existence of phylosymbiosis in fish and their microbiomes will provide insight into factors governing the microbial community associated with fish and their co-evolutionary dynamics with teleosts in general.

## Materials and methods

### Study sites

#### Detroit river

The Detroit River of the LGLs is a 51-km channel that comprises the lower portion of the Huron-Erie Corridor, connecting Lake St. Clair to Lake Erie [35]. Samples were collected around Fighting Island (from 42° 10′ 56.5″ N 83° 06′ 39.9″ W up to 42° 14′ 04.2″ N 83° 06′ 32.1″ W) from July 17 to August 29, 2018.

#### Lake Erie

Samples came from the western basin of Lake Erie, a shallow (mean 6 m), warm, and productive basin (area = 3473 km^2^) fed by the Detroit River and several smaller tributaries draining agricultural watersheds. Samples were collected on October 18, 2018.

#### Lake Ontario

Fish were collected from the Bay of Quinte and the eastern basin of Lake Ontario from July 31 to August 1, 2018. The Bay of Quinte is a large (254 km^2^), Z-shaped embayment with a history of nutrient and anthropogenic stress that feeds into the eastern basin and the upper St Lawrence River. The eastern basin is a mildly eutrophic, bathymetrically complex outlet basin of Lake Ontario with depths averaging ~ 20 m.

### Sample collection

#### Detroit River

Fish were captured using a single anode boat electrofisher (Smith-Root 5.0 GPP) set to use pulsed DC current at 60 Hz using between 30 and 60% of the range to maintain a current of 6–8 A. All fish were euthanized with an overdose of tricaine methanesulfonate (MS222) following the protocols of the Ontario Ministry of Natural Resources and Forestry (MNRF), and Canadian Council on Animal Care. The skin swabs for all fishes were immediately taken after capture by gently rubbing a sterile cotton swab over ~ 50% of the total surface on the right side of each fish. Dorsal, ventral, and pectoral fin areas were swabbed for larger fish. Swab samples were placed into 2-mL tubes and were stored on ice and transported to the Great Lakes Institute for Environmental Research (GLIER) at the University of Windsor and immediately frozen at − 20 °C. The whole fish were transported on ice with the swabs to GLIER, and within 2–4 h after capture, the fish were dissected, and gut content samples were collected and stored frozen at − 20 °C. Water samples (500 mL) were collected at the sampling sites and transported to GLIER on ice for filtration. Water samples were filtered using 0.22-μm pore size, 47-mm-diameter polycarbonate membranes (Isopore™, Millipore, MA), and stored at − 20 °C until DNA extraction.

#### Lake Erie

Fish were captured using bottom-set, graded mesh (32- to 152-mm) stretch monofilament gillnets. After capture, all fish were euthanized in compliance with the protocols of the MNRF and Canadian Council on Animal Care. The skin, fish, and water sampling, as well as the transportation and storage of the samples, were the same as for Detroit River.

#### Lake Ontario

Fish were captured using bottom-set, graded mesh (38–152 mm) stretch monofilament gillnets. Upon capture, all fish were euthanized following the protocol described for Detroit River and Lake Erie. Skin swab samples and gut tissue (foregut, midgut, and hindgut) with content were taken at the Glenora Fisheries Station, Ontario, Canada. Samples were stored in a 50 mL falcon tubes filled with 45 mL of a high salt solution (700 g/L ammonium sulfate, 25 mM sodium citrate, 20 mM ethylenediaminetetraacetic acid, pH 5.2) for 48 h to let the salts penetrate the samples (swabs, and gut content) and then stored at − 20 °C until DNA extraction. Water samples (500 mL) were collected and filtered at the Glenora Fisheries Station by using 0.22 μm filters pore size, 47 mm diameter, and the filters kept in high salt solution until delivered to GLIER. All samples were stored at − 20 °C until DNA extraction.

### Sample information

A total of 334 fish from Detroit River (*n* = 98, 29%), Lake Erie (*n* = 90, 27%), and Lake Ontario (*n* = 146, 44%) representing 17 different species were collected for microbiome characterization. The fish species included herbivores, invertivores, piscivore, planktivores, planktivore/invertivore, invertivores/carnivores, invertivores/detritivores, and invertivores/herbivores (Table 1; Supplementary Table S1) [36, 37]. For each of the 334 fish, fork length and total weight were recorded. Gut samples were taken after carefully dissecting the fish with a new razor blade or sterilized scissors to isolate a section comprising midgut and hindgut with both tissue and gut content. For Detroit River, Lake Erie, and Lake Ontario (based on sampling locations), a total of 4, 3, and 5 water samples were collected at the site of capture, respectively.

**Table 1:** Summary of Great Lakes fish species gut, skin, and water samples. We provide a description of taxonomy and feeding guild of the host species as well as where the fish were sampled. The number of samples included in our analyses is shown for both skin and gut samples (and total number for each fish species) after quality filtering and rarefaction

Genus	Species	Fish species (common name)	Feeding	Habitat	Detroit River		Lake Erie		Lake Ontario		T
					G	S	G	S	G	S	
*Alosa*	*pseudoharengus*	Alewife	Planktivore	Pelagic	-	-	-	-	20	21	41
*Aplodinotus*	*grunniens*	Freshwater Drum	Invertivore	Benthic	1	5	17	16	10	10	59
*Ambloplites*	*rupestris*	Rock Bass	Invertivore	Benthopelagic	3	7	-	-	3	3	16
*Catostomus*	*commersonii*	White Sucker	Invertivore/detritivore	Benthic	5	9	-	-	2	2	18
*Cyprinella*	*spiloptera*	Spotfin Shiner	Invertivore/herbivore	Benthopelagic	12	11	-	-	-	-	23
*Dorosoma*	*cepedianum*	American Gizzard Shad	Herbivore	Pelagic	9	10	-	-	2	2	23
*Labidesthes*	*sicculus*	Brook Silverside	Planktivore / invertivore	Pelagic	7	7	-	-	-	-	14
*Lepomis*	*gibbosus*	Pumpkinseed	Invertivore	Benthopelagic	5	10	-	-	5	5	25
*Morone*	*americana*	White Perch	Invertivore/carnivores	Benthopelagic	-	-	21	21	11	12	65
*Morone*	*chrysops*	White Bass	Invertivore	Benthopelagic	-	-	10	10	10	11	41
*Neogobius*	*melanostomus*	Round Goby	Invertivore	Benthic	-		8	8	16	16	48
*Notropis*	*atherinoides*	Emerald Shiner	Planktivore	Benthopelagic	5	15	-	-	-	-	20
*Notropis*	*heterolepis*	Blacknose Shiner	Invertivore/herbivore	Benthopelagic	11	8	-	-	-	-	19
*Perca*	*flavescens*	Yellow Perch	Invertivore	Benthopelagic	10	10	15	15	15	16	81
*Salmo*	*trutta*	Brown Trout	Piscivore	Benthopelagic	-	-	-	-	16	16	32
*Salvelinus*	*namaycush*	Lake Trout	Piscivore	Benthopelagic	-	-	-	-	22	22	44
*Sander*	*vitreus*	Walleye	piscivore	Benthopelagic	-	-	19	19	6	7	51
-	-	Water samples	-		3	-	2	-	5	-	10

*G* Gut, *S* Skin, *T* Total number of fish

### DNA extraction, library construction, and sequencing

DNA from all samples were extracted using a sucrose lysis buffer protocol as previously described [38]. The V5 (787 F-acctgcctgccg-ATTAGATACCCNGGTAG) and V6 (1046 R-acgccaccgagc-CGACAGCCATGCANCACCT) variable regions of the 16S rRNA were selected for PCR amplification. The first and second PCR conditions were same as previously published methods [38]. Briefly, the PCR protocol for the first round PCR consisted of 95 °C for 3 min followed by 28 cycles of 94 °C for 30 s, 55 °C for 30 s, and 72 °C for 1 m, and a final step at 72 °C for 10 m. For each 96 well PCR plate, one negative control consisting of PCR mix with ultra-pure water instead of DNA template was included. After the first-round PCR amplification, the results were verified by visualizing amplicons on an agarose gel. After checking and verifying first-round PCR products, the PCR was purified using Sera-Mag Magnetic Beads (GE, Healthcare Life Science, UK). A short-cycle second round PCR was conducted on the purified PCR products to ligate the adaptor and barcode (10–12 bp) sequences necessary for sample identification and sequencing. The second PCR was set at 95 °C for 3 min, then 8 cycles of 94 °C for 30 s, 60 °C for 30 s, and 72 °C for 1 min, and final extension at 72 °C for 7 min. The second round PCR amplifications were visualized on an agarose gel and amplicons were combined in proportions based on their estimated concentration (between 1 and 5 μL for samples with strong clear (1 μL) to faint bands (5 μL)). Subsequently, the combined samples were gel extracted from an agarose gel and cleaned and purified using QIAquick Gel Extraction Kit (QIAGEN, Toronto, ON, Canada). In total, 299 (89%), 330 (99%), and 10 (83%) samples for gut, skin swab, and water were amplified successfully and included in the sequencing library. We also included eight PCR blanks (one for each 96 PCR plate) in our library. The concentration of purified PCR product mix (library) was measured on an Agilent 2100 Bioanalyzer with a High Sensitivity DNA chip (Agilent Technologies, Mississauga, ON, Canada). The library concentration was then diluted to 60 pmol·μL^−1^ and sequenced on an Ion S5™ sequencing system using the Ion S5™ sequencing reagents and an Ion 530™ Chip (Thermo Fisher Scientific, ON, Canada).

### Processing of 16S sequences

Two FASTQ files (one for gut and water samples and one for swab samples) were analyzed using the Quantitative Insights Into Microbial Ecology (QIIME2-2020.11) platform [39]. The FASTQ sequence files were demultiplexed using the *cutadapt demux-single* command to remove sample barcode and primer sequences. Additionally, *cutadapt trim-single* was used to identify and remove the sequencing adapters for the demultiplexed data [40]. The DADA2 pipeline (*dada2 denoise-pyro*) was used to denoise single-end sequences, dereplicate, and filter chimeras, followed by Amplicon Sequence Variant (ASV) picking [41]. Chimeric sequences were removed using the *removeBimeraDenovo* function with the “consensus” method using the default values, except the read truncation length was set to 270 (*p-trunc-len 270*). The two ASV tables and representative sequences were merged using *feature-table merge* and *feature-table merge-seqs*, respectively. Taxonomic classification was performed using the *feature-classifier* plugin [42] and the SILVA 138–99 reference database [43]. This plugin supports taxonomic classification of features using the Naive Bayes method. All ASVs were aligned with mafft [44] (via *phylogeny align-to-tree-mafft-fasttree* command) and used to construct a phylogeny with fasttree [45]. After quality control, chimera removal and combining the two feature tables, the table was summarized with the *feature-table summarize* command. A total of 18,147,574 sequences were obtained for 647 samples (299 gut samples, 10 water samples, 330 skin samples plus eight negative controls). The eight negative controls had zero to 10 sequence reads with no consistent taxa present. The *decontam* [46] (version 1.8.0) package in the R was used to identify the blank sample ASVs as possible contaminants in our sample microbiota; however, none of the blank sample ASVs were identified as a contaminant. Thus, the negative controls were excluded from the rest of the study and we assumed contamination was not an issue for our data set.

We used the *taxa filter-table*, to remove ASVs related to mitochondria, chloroplast, and eukaryote sequences (3% of total sequence). We also removed “Unassigned” ASVs (13%), as well as bacteria and archaea ASVs without phylum assignment (1%), resulting in a total of 14,990,847 sequences remaining. The ASV table was rarefied to 3000 reads per sample for the alpha and beta diversity estimation because most of the rarefaction curves plateaued at ~ 3000 reads. Samples with fewer than 3000 reads were deleted. These deleted samples included three gut and six swab samples (Lake Trout (*Salvelinus namaycush*) (1 gut, 2 swab samples), White Perch (*Morone americana*) (1 gut), White Bass (*Morone chrysops*) (1 gut), Blacknose Shiner (*Notropis heterolepis*) (1 swab), Pumpkinseed (*Lepomisgibbosus*) (1 swab), White Sucker (*Catostomus commersonii*) (1 swab), Freshwater Drum (*Aplodinotus grunniens*) (1 swab)). This decreased the total number of samples to 630 samples (296 gut, 324 swab and 10 water samples) (Table 1). ASVs were retained for further analysis only if they had at least 10 sequence reads in at least two samples. So, the final analysis included 13,884,500 reads (93% of reads were retained) and 6,597,ASVs (15% of total ASVs (43,763)).

### Bacteria alpha and beta diversity

Bacteria alpha diversity indices were calculated for each sample using QIIME alpha diversity alpha command. The calculated alpha diversity indices were Chao1 (a metric for species richness), and Faith’s phylogenetic diversity (PD) (a metric that incorporates both species richness and species evenness), Shannon entropy. We estimated beta diversity as the Bray–Curtis dissimilarity matrix among all samples. The rarefied ASV table was used for all subsequent analyses, unless stated. Raw data are available at the sequence read archive of NCBI with PRJNA701818 BioProject accession number.

### Statistical analysis of sequence variants

#### Fish versus environmental (water) microbiota

To test for the effect of the environmental (water) microbiota on gut and skin microbiota, taxonomical compositions of the microbiota from the different sample types (gut, skin and water) were visualized using stacked barplots of the relative abundance of the bacteria at the phylum and family level with the online tool MicrobiomeAnalyst [47]. Subsequently, a non-metric multidimensional scaling (NMDS) plot using the Bray–Curtis distance matrix was generated using *vegan* (v.2.6–4) [48] and *ggplot2* (v.3.3.5; [49]) packages in R (v.4.1.0; [50]) to visualize clustering among the sample types (skin, gut, and water). We then performed permutational analyses of variance (PERMANOVA) using *adonis2* in the vegan (v.2.6–4) [48] package in R [50] to test for significant differences in beta diversity among the sample types (gut vs water, as well as skin vs water samples). Finally, differences in alpha diversity (species richness and evenness (Shannon entropy, Chao1, PD)) among the different sample types (gut vs water, as well as skin vs water samples) at all locations combined as well as within each location separately were tested using the Kruskal–Wallis (KW) rank test, followed by a post hoc Dunn test with Bonferroni corrected *P* values in SPSS (IBM SPSS 25).

#### Fish gut versus skin microbiota

Taxonomic composition of the microbiota of gut and skin samples from seventeen fish species sampled at three locations (Detroit River, Lake Erie, and Lake Ontario) were visualized using stacked barplots of the relative abundance of the bacteria at the family level using *phyloseq* [51] and *ggplot2* [49] packages built on R [50]. Bacteria families with relative abundance less than 10% (ranging between 0 and 9.99%) in all samples were combined and presented as “Family < 10 percent” in barplots. To test for the differential abundance of bacterial taxa between the gut and skin samples, the ASV table data were aggregated to the family level in R (v.4.1.0) using *phyloseq* package [51]. Subsequently, the non-normalized ASV table was used for the negative binomial Wald test in DESeq2 [52]. We used the default DESeq2 settings with negative binomial generalized linear model (GLM) fitting with Wald significance tests. *P*-values were adjusted for multiple testing using the Benjamini–Hochberg false discovery rate correction (FDR) [53]. Bacterial taxa showing differential abundance were defined with the thresholds of FDR < 0.05 and |log_2_ Fold Change (FC)|> 2. Differences in alpha diversity (Shannon entropy, Chao1, PD) between gut and skin samples were statistically tested using the KW rank test (as described in Fish versus Environmental (Water) microbiota section above). To test for significant beta diversity differences between gut and skin samples, we used PERMANOVAs analysis with *adonis2* in *vegan* (v.2.6–4) [48] package in R [50].

#### Endogenous and exogenous factor analyses

Gut and skin samples clustering based on location and fish species were visualized using an NMDS plot. Observed differences were assessed for significance with PERMANOVA analyses using *adonis2* in *vegan* (v.2.6–4) [48]; however, we only included samples where fish species occurred in at least two of the three locations. We explored the role of location, fish species, body weight, and the interaction of fish species with sampling location in our PERMANOVA model for both gut and skin samples. Subsequently, we used least absolute shrinkage and selection operator (known as LASSO regression) in R in the *glmnet* package (v.4.1.7) [54] to identify the variables and corresponding regression coefficients that lead to a model that minimizes the prediction error and results in variable selection when there is a high likelihood of multicollinearity. We first quantified beta diversity in the gut and skin microbiota using a principal coordinates analysis (PCoA) in R (v.4.1.0) and retained the first five PCoA axes values (gut sample PCoA factors retained = eigenvalue > 2, percent of variation > 6%; skin sample PCoA factors retained = : eigenvalue > 5, percent of variation > 3%). We used Shannon entropy, Chao1, and PD (as described above) to quantify alpha diversity.

Given that we found evidence for significant species, location, and interaction effects on both gut and skin microbiome alpha and beta diversity, we included further analyses of possible mechanisms driving those effects. Specifically, the location effect may reflect habitat preferences among the 17 fish species sampled and the species effect may reflect diet preferences among the sampled species. To explore fish species, diet, and habitat preference effects on gut and skin bacterial community composition (alpha (Shannon entropy, Chao1 and PD) and beta diversity indices (PCoA1-5)), we built several linear mixed-model (LMM) models in R (v.4.1.0) using the *lme4* [55] package with fish species, habitat, diet, and body weight as fixed factors and location as a random factor. For selecting the best model for each diversity index, we used *AICcmodavg* package (v. 2.3.2) [56] in R (v.4.3.1) using Akaike Information Criterion (AIC) after building the models. We log_10_ transformed weight, Shannon entropy, Chao1, and PD to meet the normality and heteroscedasticity assumptions of LMMs.

#### Host-microbiome phylosymbiosis

To test if skin and gut microbiota composition were linked with host phylogeny, cytochrome c oxidase I (*COX1*) and cytochrome b (*cytb*) sequences for the 17 host fish species were downloaded from the NCBI website (https://www.ncbi.nlm.nih.gov/gene) and combined to create an artificial concatenated sequence. The concatenated sequences were aligned using MUSCLE [57] on the CIPRES Science Gateway v.3.1 [58]. Subsequently, pair-wise phylogenetic distances between the species were calculated using the Kimura two-parameter model of substitution in MEGA-X (version 10.2.6) [59]. Finally, to evaluate the effect of host phylogeny on microbiome dissimilarity (phylosymbiosis), we performed Mantel tests (*method* = *"pearson", permutations* = *999, na.rm* = *TRUE)* in R (version 4.3.1) using the *vegan* (V2.6–4) package [60] to compare the bacterial community Bray–Curtis dissimilarity matrixes (skin and gut) and fish species phylogenetic distance matrix. To do so, Bray–Curtis dissimilarity was calculated across all samples within each fish species.

## Results

### Fish versus environmental (water) microbiota

Taxonomic composition of the skin, gut, and water microbiota were distinct; however, the water microbiota was most divergent. Across all fish species, the fish microbiota were dominated at the phylum level by Pseudomonadota (previously known as Proteobacteria) (78% (skin), 56% (gut)), Fusobacteriota (6% (skin), 19% (gut)), and Bacillota (previously known as Firmicutes) (7% (skin), 18% (gut)). However, the water sample taxa showed a different set of common taxa, with only Pseudomonadota in common among the fish bacteria taxa (Pseudomonadota (38%), Actinobacteriota (27%), Bacteroidota (18%)) (Fig. S1). At the family level, the fish microbiota were dominated by members of *Aeromonadaceae* (gut (21%), skin (19%)), *Fusobacteriaceae* (gut (19%), skin (6%)), *Enterobacteriaceae* (gut (17%), skin (17%)), and *Moraxellaceae* (gut (1%), skin (20%)) (Fig. S2). However, at the family level, the water microbiota was dominated by *Comamonadaceae* (23%), *Sporichthyaceae* (19%), and *Chitinophagaceae* (5%) (Fig. S2).

**Fig. 1:**
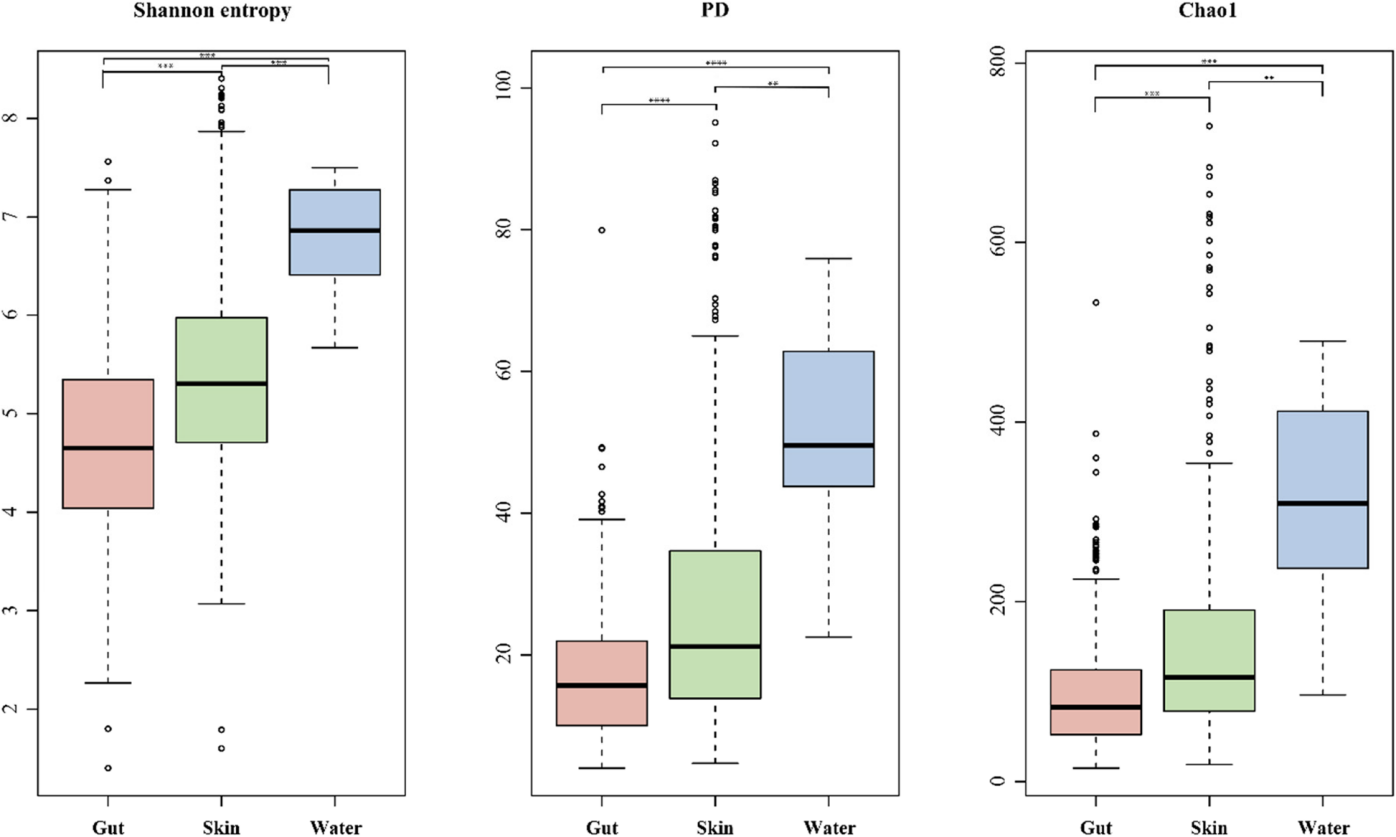
Box and whisker plots of alpha diversity indices (Shannon entropy, Faith’s PD, and Cho1) for gut, skin, and water microbiomes. The colors reflect the sample type and the black circles are outliers. The black line in each box plot is the median. Stars indicate significance level (significance codes: 0.001 < *P* ≤ 0.01**, *P* ≤ 0.001***)

Measures of alpha diversity (Shannon entropy, PD, Chao1) showed higher diversity in the water microbiota than in the fish gut and skin microbiota (Shannon entropy: KW 81, *P* < 0.0001; PD: KW 74, *P* < 0.0001; Chao1: KW 63, *P* < 0.0001;) (Fig. 1, Table 2). The post hoc pairwise comparisons revealed that when water samples were compared against gut and skin samples, gut sample microbiota were more divergent (Shannon entropy: test statistic − 320, adj *P* < 1.38E − 7; PD: test statistic − 316, adj *P* < 1.97E − 7; Chao1: test statistic − 292, adj *P* < 2.00E − 06) than the skin microbiota (Shannon entropy: test statistic − 205, adj *P* = 0.001; PD: test statistic − 208, adj *P* = 0.001; Chao1: test statistic − 192, adj *P* = 0.003) (Fig. 1, Table 2). We also tested for differences between gut vs water, and skin vs water separately within each location. The differences between gut vs water compared to skin vs water were more pronounced when we performed the analysis within each location (Table 2).

**Table 2:** Results of the Kruskal–Wallis *H* test followed by a post hoc Dunn test testing differences in alpha diversity indices (Shannon entropy, Faith’s phylogenetic diversity (PD), and Chao1) for gut, skin, and water samples among all locations followed by separate tests within each location

Variables	Diversity index	Post hoc pairwise comparisons	Test statistic	Std. error	Adj. Sig.^a^	*d*	Kruskal–Wallis *H*
All locations	Shannon entropy	Gut-skin	− 114	15	1.39E − 14	2	81 ***
		Gut-water	− 320	58	1.38E − 7		
		Skin-water	− 205	58	0.0007		
	PD	Gut-skin	− 108	14.6	4.69E − 13	2	74 ***
		Gut-water	− 316	58.5	1.97E − 7		
		Skin-water	− 208	58.4	0.0014		
	Chao1	Gut-skin	− 99	14.6	2.94E − 11	2	63 ***^b^
		Gut-water	− 292	58.5	2.00E − 6		
		Skin-water	− 192	58.4	0.003		
Lake Ontario	Shannon entropy	Gut-skin	− 58	10	8.77E − 9	2	47 ***
		Gut-water	− 160	37.6	6E − 5		
		Skin-water	− 101	37.6	0.02		
	PD	Gut-skin	− 62	9.8	7.14E − 10	2	52 ***
		Gut-water	− 160	37.7	5.00E − 5		
		Skin-water	− 98	37.7	0.02		
	Chao1	Gut-skin	− 36	9.8	7.00E − 4	2	23 ***
		Gut-water	− 134	37.7	0.001		
		Skin-water	− 98	37.7	0.02		
Lake Erie	Shannon entropy	Gut-skin	− 58	8	8.01E − 14	2	57 ***
		Gut-water	− 83	37.4	0.02		
		Skin-water	− 25	37.4	0.51		
	PD	Gut-skin	− 72	7	0.00E0	2	88 ***
		Gut-water	− 97	37	0.02		
		Skin-water	− 24	37	1.00		
	Chao1	Gut-skin	− 67	7	0.00E0	2	76 ***
		Gut-water	− 85	37	0.06		
		Skin-water	− 18	37	1.00		
Detroit River	Shannon entropy	Gut-skin	− 1.5	7.5	1.00	2	7*
		Gut-water	− 76	28	0.02		
		Skin-water	− 75	28	0.02		
	PD	Gut-skin	17	7	0.05	2	13**
		Gut-water	− 66	27	0.05		
		Skin-water	− 84	27	0.007		
	Chao1	Gut-skin	7	7	1	2	8*
		Gut-water	− 69	27	0.03		
		Skin-water	− 76	27	0.01		

^a^Significance values have been adjusted by the Bonferroni correction for multiple tests

^b^Significance codes: 0.01 < *P* ≤ 0.05*, 0.001 < *P* ≤ 0.01**, *P* ≤ 0.001***

**Fig. 2:**
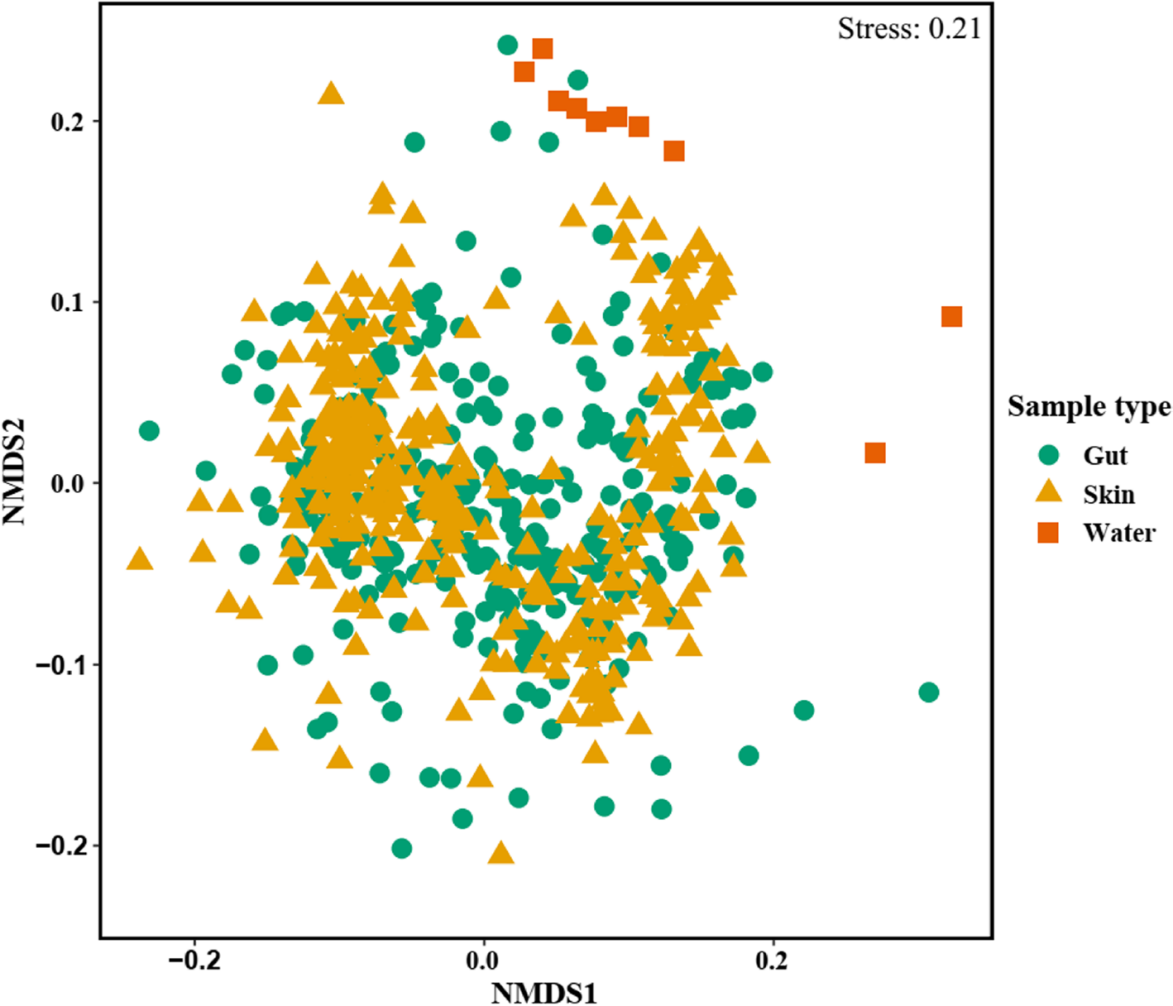
NMDS plot of microbiota associated with fish gut, skin, and water samples based on Bray–Curtis dissimilarity matrix. Shapes and colors are based on sample types

The NMDS plot also showed clear separation between the fish microbiota and the water microbiota (Fig. 2). The gut and skin microbiota showed considerable overlap in the NMDS; however, the water microbiota clustered separated from the fish microbiota, indicative of different community composition (Fig. 2). PERMANOVA analyses confirmed the statistical significance of the NMDS clusters (PERMANOVA pseudo-F: 12.8, *P* value < 0.001). Subsequently, individual PERMANOVAs for gut-water and skin-water comparisons showed significant effects for gut microbiota (*t*-value: 3.02; *p*-value < 0.001) and skin microbiota (F-value: 3.01; *p*-value < 0.001) compared to water microbiota.

### Fish gut versus skin microbiota

**Fig. 3:**
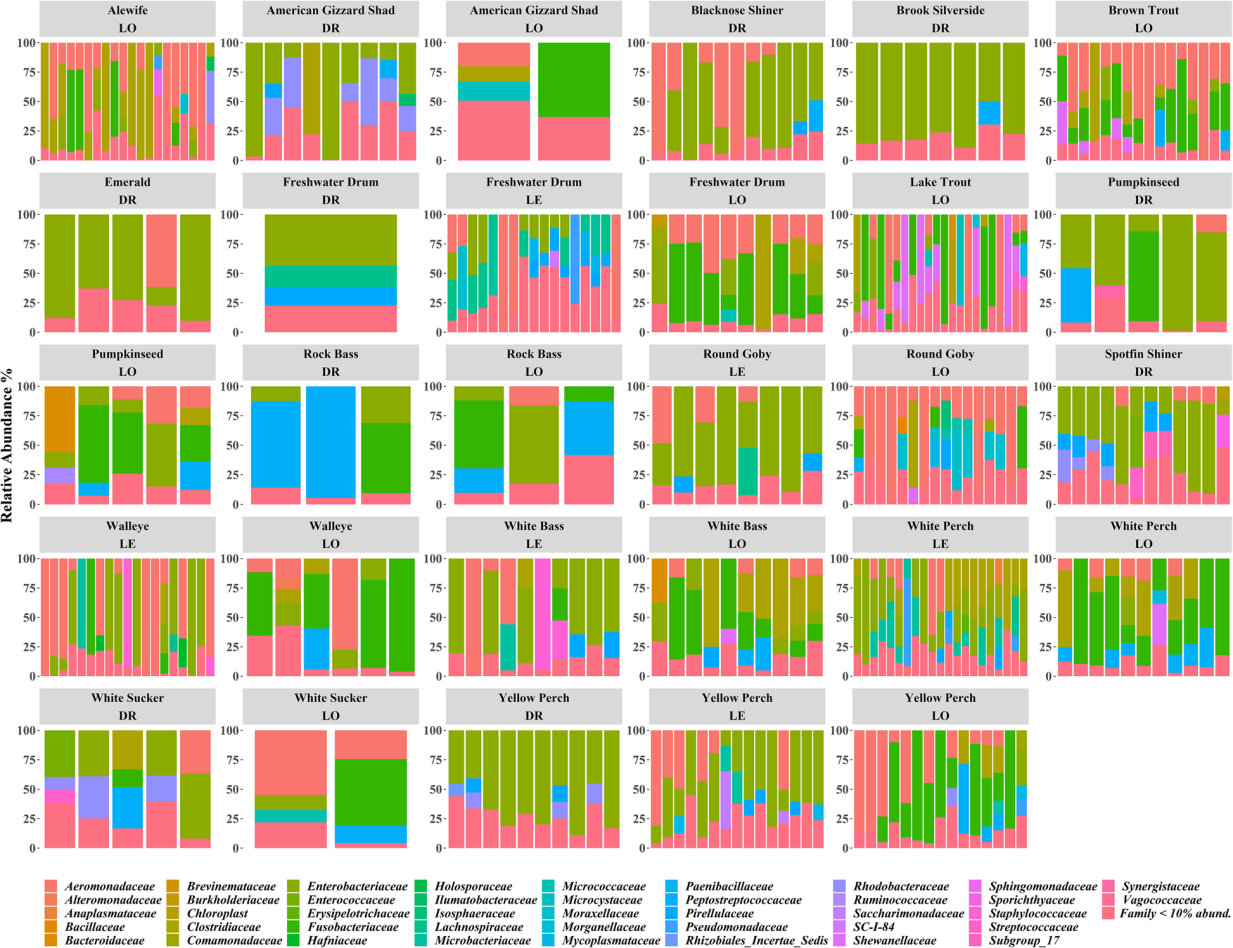
Stacked bar plots showing the relative abundance of gut bacterial community composition presented at the family level for all 17 fish species at the three sample locations (DR, Detroit River; LE, Lake Erie; LO, Lake Ontario). Each bar is representative of an individual fish within that species

**Fig. 4:**
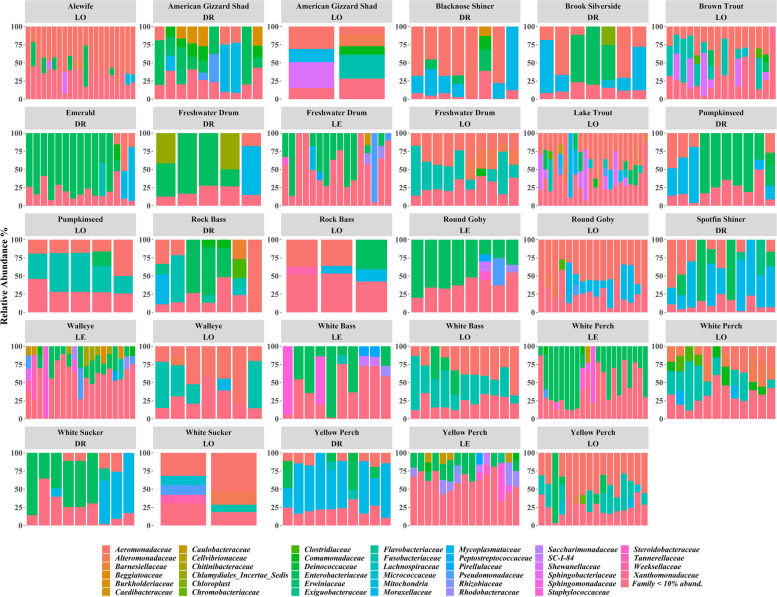
Stacked bar plots showing the relative abundance of skin bacterial community composition presented at the family level for all 17 fish species at the three sample locations (DR, Detroit River; LE, Lake Erie; LO, Lake Ontario). Each bar is representative of an individual fish within that species

Taxonomic analysis of the microbiota of skin and gut showed that Pseudomonadota (Fig. S1), and specifically members of the *Aeromonadaceae* and *Enterobacteriaceae* families generally dominated the fish microbiota (Figs. 3 and 4 and Fig. S2). However, gut and skin samples exhibited different bacterial taxa (Figs. 3 and 4). Differential abundance analysis at the family level was used to acquire more specific insight into differences in microbiota composition between the gut and skin microbiota. Comparing bacterial abundance data at the family level between skin and gut identified 37 bacteria families that had statistically significant differences (Table S2). For example, while members of the *Deinococcaceae*, *Exiguobacteraceae*, and *Moraxellaceae* families were in high abundance in skin samples, they were rare in the gut sample microbiota. On the other hand, *Microbacteriaceae* and *Lachnospiraceae* were at higher abundance in the gut microbiota (Table S2).

Moreover, the alpha diversity comparisons between gut and skin samples showed that skin samples had higher diversity relative to gut samples (Shannon entropy: test statistic − 114, adj *P* < 1.39E − 14; PD: test statistic − 108, adj *P* < 4.69E − 13; Chao1: test statistic − 99, adj *P* < 2.94E − 11) (Table 2, Fig. 1). Although gut and skin samples showed considerable overlap in the NMDS plot (Fig. 2), our PERMANOVA analysis showed that gut and skin samples had significantly different microbiota based on Bray–Curtis dissimilarity (*t*-value: 4.08; *p*-value < 0.001).

### Endogenous and exogenous factor analyses

**Fig. 5:**
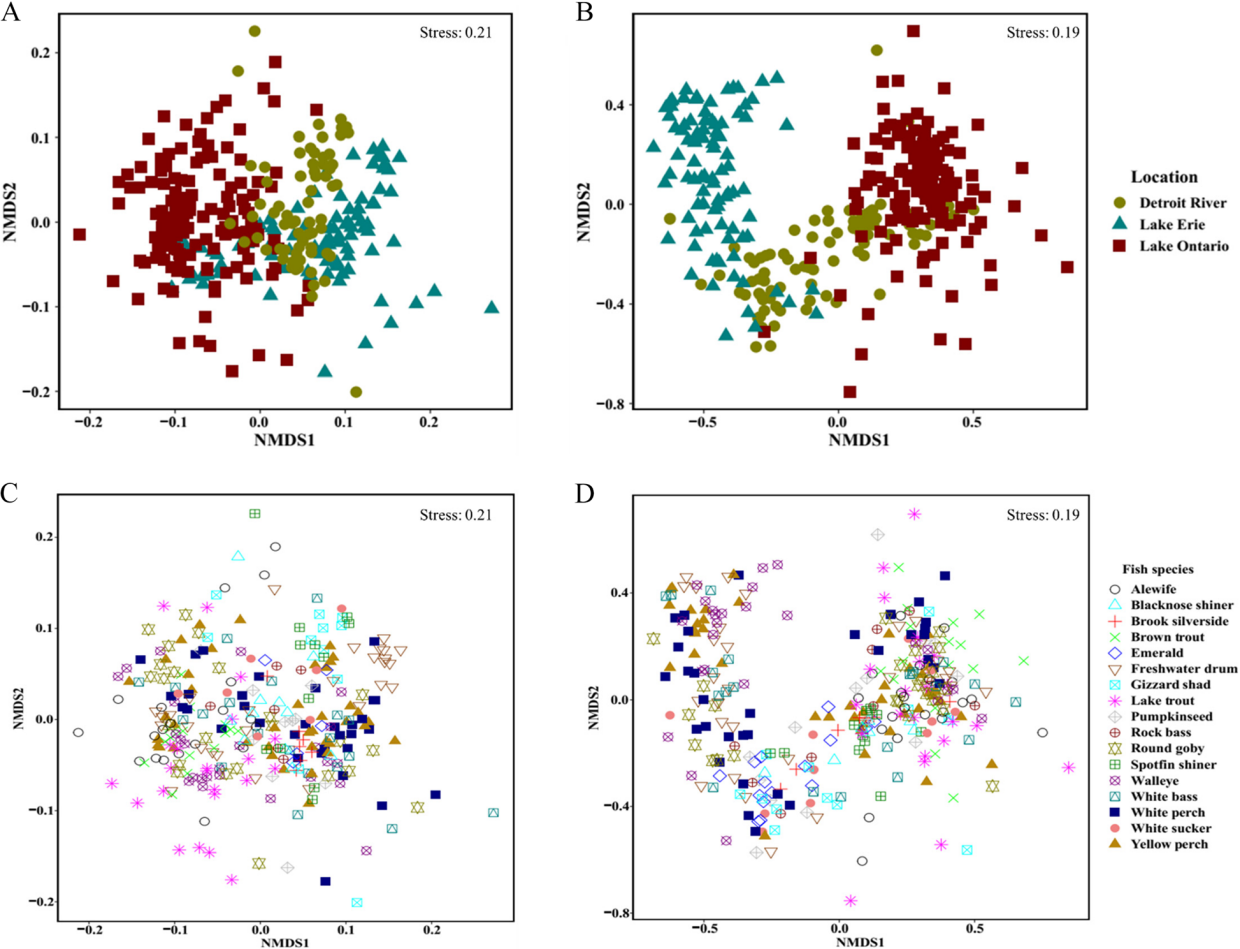
NMDS scatter plots of gut and skin microbiome bacterial community composition based on Bray–Curtis distance matrices for 17 species of fish sampled at three locations (Detroit River, Lake Erie, and Lake Ontario). The left panels **A** and **C** show the NMDS for the gut microbiome bacterial community compositions, while the right panels **B** and **D** show the NMDS for the skin microbiome bacterial communities in the same fish. The NMDS plots **A** and **B** are coded to show the location of capture for each fish; the panels **C** and **D** are coded to show the species of each fish sampled

The microbiota composition in the gut and skin samples of different fish species sampled at different locations often showed high levels of variation among and within host species and among locations, highlighting the potential effect of location and fish species on fish microbiota (Figs. 3 and 4). Furthermore, NMDS analysis showed that microbiota composition clustered based on the host fish location (Lake Erie, Lake Ontario, Detroit River) (Fig. 5A, B) and host fish species within the location for both gut and skin microbiota (Fig. 5C, D). PERMANOVA analyses supported those clustering patterns and showed highly significant effects of location, host fish species, and the interaction between host fish species and location on both the gut and skin microbiota (Table 3). Moreover, body weight also showed a significant effect on both skin and gut samples.

**Table 3:** PERMANOVA results for the effects of sample location, fish species, and their interaction on bacterial community beta diversity (Bray–Curtis dissimilarity matrix) for both skin and gut samples. Only fish species captured at two or more locations were included in this analysis

Variables	df	SS	*R*^2^	*F* value
**Gut**				
Location	2	10.5	0.13	17***
Fish species	9	6.6	0.08	2.4***
Weight	1	0.65	0.008	2.1**
Fish species × location	10	6.3	0.07	2***
Residuals	180	55.7	0.70	
Total	202	79.2	1.00	
**Skin**				
Location	2	15.2	0.17	26***
Fish species	9	5.9	0.06	2.2***
Weight	1	0.78	0.009	2.7**
Fish species × location	10	6.3	0.07	2.1***
Residuals	201	59	0.68	
Total	223	86.6	1.00	

Significance codes: 0.001 < *P* ≤ 0.01**, *P* ≤ 0.001***

Our LASSO regression analysis showed that the best predictor variables for gut samples were different based on the diversity indices (alpha (Shannon entropy, PD, Chao1) and beta diversity (PCoA 1- 5)), with fish species identified as the least predictor for alpha diversity indices and diet and location as the best predictor (Fig. S3). On the other hand, fish species and locations were the best predictors for beta diversity indices. For skin samples, both alpha and beta diversity indices showed fish species followed by weight as the best predictors (Fig. S4). Our LMM analysis (with fish species, habitat, and diet included as fixed factors and location as a random factor) of the effects on bacterial diversity indices (alpha (Chao1, PD) and beta (PCoA 1–5)) showed that fish species, diet, and habitat had significant effects on alpha diversity indices for gut samples but not for skin samples (Table 4). Moreover, fish species, diet, and habitat had significant effects for both gut and skin samples for the beta diversity indices. Despite including diet and habitat in our model, fish species remained as a significant effect, indicating the species effect is likely more complex than simple species-specific diet or habitat preferences.

**Table 4:** Summary of the LMM outcomes testing for the effects of host fish species, habitats, and diets on alpha diversity (Chao1, Shannon entropy, PD) and beta diversity (PCoA 1–5) of the gut and skin microbiome bacterial communities across 17 species of fish and three habitats of the Great Lakes

Model	Variables	Sum Sq	Mean Sq	*F* value
**Gut samples**				
**Shannon entropy** ~ Habitats + Diet + Fishname + (1|Locations), REML = FALSE	Diet	25.6	3.65	4.26***
	Habitats	6.2	3.10	3.61*
	Fish species	16.4	1.64	1.91*
** Chao1** ~ Habitats + Fishname + (1|Locations), REML = FALSE	Habitats	4.7	0.27	4.44***
	Fish species	1	0.51	8.25***
** PD** ~ Fishname + (1|Locations), REML = FALSE	Fish species	3.3	0.17	3.97***
** Axis.1** ~ Habitats + Diet + Fishname + (1|Locations), REML = FALSE	Diet	3630	518	1.98
	Fish species	13,546	1354	5.17***
	Habitats	1095	547	2.09
** Axis.2** ~ Diet + (1|Locations), REML = FALSE	Diet	11,736	1676	7.56***
** Axis.3** ~ Habitats + Fishname + (1|Locations), REML = FALSE	Habitats	2686	1343	9.97***
	Fish species	1108	651	4.84***
** Axis.4** ~ Fishname + (1|Locations), REML = FALSE	Fish species	16,933	891.22	3.40***
** Axis.5** ~ Fishname + (1|Locations), REML = FALSE	Fish species	5303	279.14	3.27***
**Skin samples**				
**Shannon entropy** ~ Diet + (1|Locations), REML = FALSE	Diet	6.3	0.79	0.81
**Chao1** ~ Fishname + (1|Locations), REML = FALSE	Fish species	1.2	0.07	1.20
**PD** ~ Diet + Fishname + Habitats + (1|Locations), REML = FALSE	Diet	0.46	0.05	1.30
	Fish species	0.44	0.05	1.36
** Axis.1** ~ Fishname + (1|Locations), REML = FALSE	Fish species	13,286	830.37	3.99***
** Axis.2** ~ Diet + Fishname + (1|Locations), REML = FALSE	Diet	4932	616.50	5.04***
	Fish species	4037	504.69	4.13***
** Axis.3** ~ Habitats + Diet + Fishname + (1|Locations), REML = FALSE)	Diet	954	744.36	6.13***
	Habitats	1904	952.19	7.85***
	Fish species	1986	331.05	2.72*
** Axis.4** ~ Fishname + Weight + (1|Locations), REML = FALSE)	Fish species	19,372	1210.8	14.32***
** Axis.5** ~ Fishname + (1|Locations), REML = FALSE)	Fish species	7985	499.06	4.92***

Significance codes: 0.01 < *P* ≤ 0.05*, 0.001 < *P* ≤ 0.01**, *P* ≤ 0.001***

### Host-microbiome phylosymbiosis

**Fig. 6:**
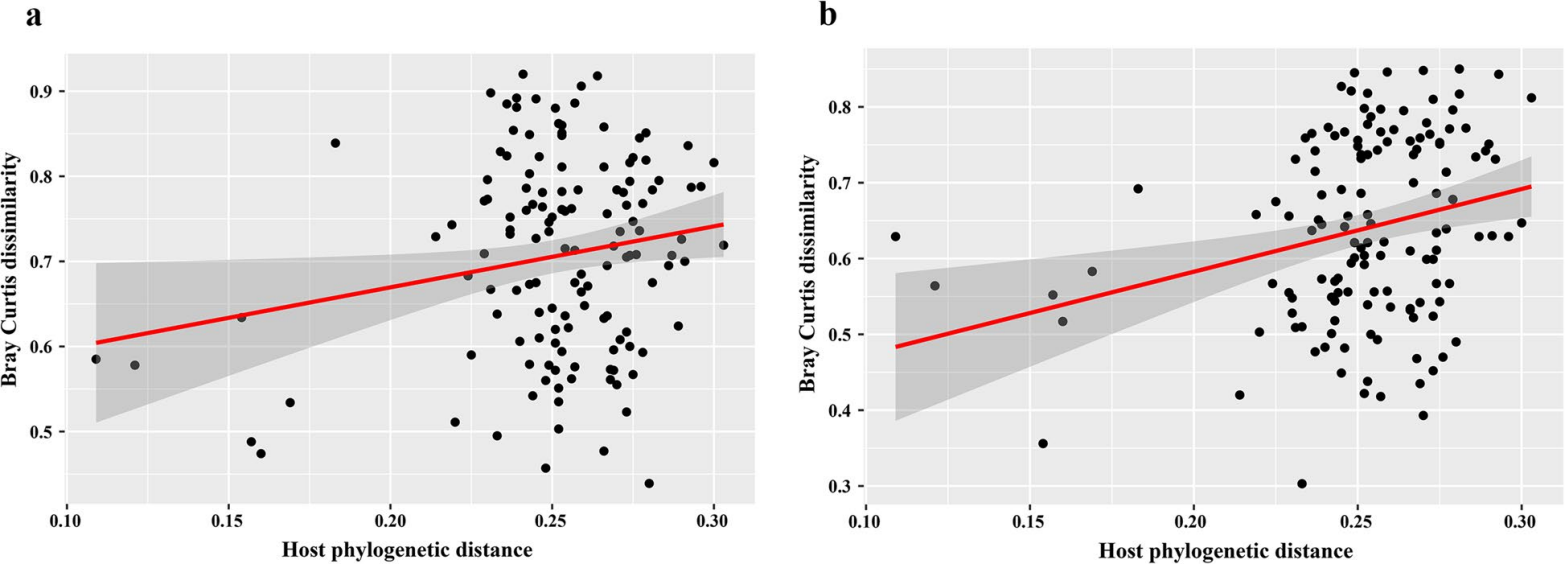
Scatterplot of pairwise host phylogenetic distance vs pairwise Bray–Curtis dissimilarity for both gut (**a**) and skin (**b**) samples. Samples were combined within host species. Host phylogenetic distance was estimated using of *CO1* and *CytB* mitochondrial gene sequences

Mantel tests of pairwise correlations between host phylogenetic distances and pairwise Bray–Curtis bacterial community dissimilarity (across all locations) values revealed a significant, but weak, positive relationship between bacterial community dissimilarity and host evolutionary distance for both gut (*r* = 0.18, *P* < 0.05) skin samples (*r* = 0.26, *P* < 0.01) supporting the phylosymbiosis for fish gut and skin samples (Fig. 6). However, as seven of the data points were separated from the rest (due to specific pylogenetic relationships—see Fig. 6), those seven data points were removed and the Mantel tests of pairwise correlations were re-calculated. By removing the seven comparisons from the data, the Mantel test revealed a significant positive relationship only between skin bacterial community dissimilarity and host evolutionary distance (Mantel statistic r: 0.25, *P* value < 0.05) (Fig. S5).

## Discussion

Diverse exogenous and endogenous factors have been reported to contribute to determining the composition of fish microbiomes [5, 14, 29]. However, the relative contributions of those factors in determining the composition of the teleost fish microbiome remain poorly characterized [7, 29]. Our study evaluated how the bacterial component of the gut and skin microbiota in 17 wild freshwater fish species sampled at three locations is shaped according to the surrounding water, trophic guild, diet, body weight, host species, and host phylogeny. While the fish microbiota (skin and gut) were different from the surrounding water microbiota, sample location (Detroit River, Lake Erie, Lake Ontario) had a strong effect on the composition of both the gut and skin microbiota across all species of fish. Moreover, host species as well as host species-by-location interactions were significant but did not explain as much variation in the fish bacterial community composition as the location’s main effect. Importantly, when we included fish diet and habitat preferences in our analysis, diet and habitat were important, but host species was still significant—indicating that the fish species effect was phylogenetically consistent and independent of diet and habitat, consistent with long-term co-evolutionary effects. When we tested the relationship between host phylogenetic distance and bacterial community dissimilarity, we found a significant correlation, consistent with phylosymbiosis. Overall, our results indicate that the host’s environment has a greater role than host-specific selection in the assembly and composition of both host-associated microbiota (gut and skin) and must be accounted for in any assessment of host-specific effects on the microbiome; however, evolutionary relationships between the host and its associated microbiomes are also important contributors.

The aquatic microbial community is thought to be the main source of the bacterial component of fish microbiomes, for both the gut and skin [61]. Nevertheless, even with the ongoing and constant exposure to the surrounding aquatic microbiome, studies indicate that fish harbor microbiome that are distinct from the water microbiome [62, 63]. Our work supports those previous findings; we showed that the microbiota in the fish gut and skin were highly distinct from the surrounding water microbiota. Similar to other studies, we found Pseudomonadota, Actinobacteriota, and Bacteroidota were the most abundant phyla in the aquatic environment [28, 29, 64]. Other studies report that Pseudomonadota, Fusobacteriota, Bacillota, and Bacteroidota often comprise up to 95% of the fish bacterial communities [6, 29, 64, 65], also consistent with our work. These divergent patterns of bacterial abundance are expected, as Pseudomonadota play an important role in the growth of fishes through nutrient cycling and the mineralization of organic compounds [66], while Bacillota and Fusobacteriota have roles in fatty acid absorption, lipid metabolism, fermentative process, and degradation of oligosaccharides in fish [27]. While, in our study, the dominant bacterial phyla in the fish had some overlap with the water microbiota, major differences were apparent, likely due to differences in the functional roles of the fish-associated versus aquatic microbiome. While differences between the fish microbiomes and the surrounding water microbiome are expected, we were surprised by the high level of divergence of the fish skin microbiota—clearly, the Teleost skin mucous microbiota plays important functional roles in the host’s performance and does not simply reflect the surrounding water’s microbiota.

While broad comparisons at the phylum level are valuable, a more detailed differential abundance analysis at the family level provides more specific insights into microbiota variation between gut and skin microbiota. Our differential abundance analysis showed fundamental differences between the microbiota of skin and gut across a diverse array of host fish species. For example, *Deinococcaceae* and *Exiguobacteraceae* were generally more abundant in the skin mucus microbiota, relative to the gut-content microbiota. On the other hand, members of *Microbacteriaceae* and *Lachnospiraceae* were more common in the gut microbiota. Bacteria in the family *Deinococcaceae* are obligate aerobes and have a high resistance to ionizing radiation (gamma- and/or ultra-violet (UV) radiation) [67]. This makes it unsurprising that *Deinococcaceae* was more abundant in the skin microbiome, as skin experiences more exposure to solar radiation than the gut. Their resistance to ionizing radiation may also contribute to *Deinococcaceae* being reported in habitats associated with high levels of solar radiation, including fish skin [68], hot springs [69], and rivers [70]. In our study, we found a general pattern of elevated levels of *Deinococcaceae* in pelagic species (such as Yellow Perch, Walleye) while lower levels were observed in deep water or benthic species (such as Brown Trout, Lake Trout, and Round Goby). The prevalence of *Exiguobacterium* in the skin microbiota in this study is consistent with its reported occurrence under a wide range of environmental conditions [71], including freshwater [72] and skin in humans [73]. Perhaps one reason for the elevated abundance of *Exiguobacterium* in fish skin samples relative to the gut samples in our study is the ability of *Exiguobacterium* to thrive under highly variable conditions [74], an important consideration for fish skin microbiome that are exposed to considerable environmental variation relative to the gut habitat. Our detection of *Microbacteriaceae* at higher levels in the gut microbiota is consistent with previous work that reported them in various terrestrial and aquatic ecosystems [75], as well as associated with fish at various life stages [76–78]. The association of *Lachnospiraceae* with the fish gut microbiota likely reflects their important functional role as intestinal symbionts of vertebrates [79], acting as butyrate producers in the intestinal microbiome. Members of *Lachnospiraceae* have been previously reported in the fish gut [80, 81] and indeed have been shown to have a symbiotic link to their host in surgeonfish [79]. Finally, we found substantially elevated levels of *Enterobacteriaceae* in the fish skin and gut samples, relative to the water samples (Fig. S1). Dominance of *Enterobacteriaceae* taxa in fish microbiomes has been widely reported in freshwater fish microbiomes [82–84] likely reflecting the importance of these bacteria for fish health and homeostasis [28, 85].

A large volume of published work shows that both endogenous and exogenous factors contribute to the Teleost microbiome composition [4, 5, 7, 8]. Endogenous factors can act at the individual level to drive variation in the microbiome (e.g., via life history, host health status, or host gene expression), as well as at the population level (e.g., via adaptation to local selection pressures) or even the species-level (e.g., via genomic variation and phylogenetic ancestry) [86]. On the other hand, exogenous abiotic (climate, water chemistry, geography, etc.) and biotic (such as water microbiome) factors can contribute to variation in microbiome composition as well [87]. Our analyses of alpha and beta diversity indices for both the skin and gut microbiota showed significant effects of location, habitat (exogenous), diet, and host fish species (endogenous). A variety of studies have reported both environmental and host species effects on the gut and skin microbiome composition in fishes [5, 29, 88, 89]. Generally, the skin microbiota is reported to be more affected by environmental factors than the gut microbiome [8, 29], which is not surprising, given the close contact between the aquatic environment and fish skin habitat [90]. Surrounding environments such as water and sediment are thought to be major sources of skin and gut microbiome [91, 92]. Moreover, we observed significant differences in bacterial diversity between skin, gut, and the surrounding aquatic environment, with fish skin and water having higher diversity than gut, similar to other studies [29, 62, 93]. This is in contrast with other studies that included humans [31] and fish [94] that showed lower diversity for the skin microbiome (although this depended on the specific measure of diversity). One reason for this could be that our fish are from wild populations and other studies often use captive fish—captivity generally reduces alpha diversity and changes the composition/structure of vertebrate skin and gut microbiomes [95]. Similarly, in humans, westernization significantly affects human microbiome diversity by lowering the diversity of bacteria [96]. In this study, sample location dominated host species effects, with, as expected, a stronger effect for the skin microbiota (*R*^2^ = 0.17) than for the gut microbiota (*R*^2^ = 0.13), although host species effects were still substantial (skin microbiota (*R*^2^ = 0.05); gut microbiota (*R*^2^ = 0.05)). This pattern of effects on skin versus gut microbiota was despite the strong microbiota divergence between both fish microbiota and the water microbiota. However, our Kruskal–Wallis results also showed that skin microbiota were more similar to that found in the water than the gut microbiota. As the skin is in constant direct contact with the surrounding water microbiome, the skin microbiome would be expected to reflect at least part of the bacteriological composition of the surrounding water [97]. By contrast, the gut microbiome is known to be strongly influenced by host-related factors and diet [98]. In this study, we included 17 different fish species sampled at three different locations, and while we found a strong host species and location effects for both gut and skin, we recognized that those relationships may actually reflect local dietary/habitat preference variation among the study species [89, 99]. Thus, our reported exogenous factor (location) may actually include a component of endogenous effects. This is further supported by the significant species-by-location interaction effect; such an effect reflects species-specific sample location effects, which would logically be due to differences in host diet, at least for the gut microbiome. Moreover, our study showed approximately equal effects of host species effects on the gut and skin microbiota, perhaps due to the inclusion of multiple host species that utilized the aquatic habitat quite differently. Our observed host fish species effects on both gut and skin microbiome microbiota agree with previous research showing interindividual, population, and species variation for microbial community composition [100], all of which were interpreted as due to host endogenous factors. Given that the gut microbiome habitat is highly controlled by the host’s physiology, only bacterial species adapted to that environment would be expected to thrive in the gut; hence, the host should have a considerable effect on the gut bacterial community composition [29]. The specific mechanisms driving variation in the fish bacterial community are still unclear, despite substantial research (including this work), likely due to complex interactions among possible mechanisms.

Microbiome similarity among species in a community is predicted to decrease with increasing evolutionary divergence of the host organisms [17], this is the basis for phylosymbiosis. However, phylosymbiotic patterns can result from diverse factors, including phenotypic divergence among fishes that are phylogenetically distant [15], co-evolution between the individual bacteria in the microbiome and the host [101], and even patterns of host behavior or life history that may be correlated with phylogeny but also indirectly affect the microbiome (e.g., feeding preferences) [22]. Additionally, evolutionary processes such as selection and drift can also shape the species relatedness and their associated bacterial community, thus resulting in phylosymbiosis [16, 102]. Numerous studies have documented an effect of the host fish species on microbiome [4, 85, 88]. We showed correlations between bacterial community composition divergence and host fish taxonomic divergence for both the gut and skin microbiota. While specifies-specific diet differences do contribute to bacterial community similarity, the species effects were still present after correcting for diet, indicating that the mechanism behind our observed phylosymbiosis cannot be due to diet alone. Previous studies showed that host-specific microbiomes are a widespread pattern in nature, occurring in many host organisms [16], including mammals [19], birds [103], insects [104], and fish [4, 94, 105]. Although some bacterial lineages may still co-diversify with hosts, it is important to note that phylosymbiosis by itself is not an indicator of host–microbiome adaptive co-evolution. Evidence for phylosymbiosis in non-mammalian vertebrate animals is incomplete and inconsistent [16]. For example, some studies in fish showed evidence for phylosymbiosis [4, 94], whereas others report mixed or weak evidence [7, 29]. Curiously, despite the fact that multiple studies (including ours) have shown that the fish skin microbiome is generally more affected by the environment than the gut microbiome [29, 62], we found a stronger signal of phylosymbiosis for the fish skin microbiota than for the gut microbiota. This was not expected, as the fish skin microhabitat is much more affected by environmental physicochemical parameters [29], not to mention the water microbiome. Clearly, the host species still has a substantial effect on even their peripheral microbiomes, highlighting the functional importance of all host-associated microbiomes. Given that our sampled fishes included 17 species representing 7 orders, our phylosymbiosis analysis is powerful and provides a solid foundation for future evaluations of the role of co-evolution between host organisms and their microbiome communities.

Overall, our findings contribute to the characterization of the modulators of microbiome composition and diversity across fish taxa. While many studies have characterized fish microbiome, yet few of those studies included multiple species sampled in the wild across multiple locations. Our study design provided a robust test of the relative effects of habitat, host diet, and host species on the bacterial communities of two key microbiomes associated with fish health and fitness. Not surprisingly, we found that the fish microbiota are distinct from the aquatic environmental microbiota, but that sampling location had a strong effect on microbiota composition, nevertheless. Curiously, we found strong host species-by-location interaction effects for both skin and gut microbiota, indicating that the species effects varied among the three sampled locations, possibly due to local fish diet and/or habitat-use differences. As expected, we also found a significant, but less strong effect of host fish species on both the gut and skin microbiota. Based on the host fish species effect that persisted after correcting for diet and habitat preferences, we tested for, and identified, significant phylosymbiotic signals between host phylogeny and both the gut and skin microbiome. This suggests that both the gut and skin microbiota co-evolved with their host species, although ecological covariation also contributes substantially since the variance in microbiota similarity explained was modest. Investigations of the nature of fish-microbe associations and, whether they are sustained, functional relationships or transient effects of fish and habitat associations are critical to further our understanding of the potential beneficial interactions between hosts and their microbiomes.

### Supplementary Information

**Additional file 1: Figure S1. **Relative abundance of bacterial community composition presented at the phylum level for gut, skin, and water microbiota (samples are combined across sample types). Phyla with less than 0.01% of relative abundance are combined and presented as “others”). **Figure S2.** Bacterial community composition (relative abundance at the family level) for gut, skin, and water microbiomes across all fish species collected at three sites in the Great Lakes (Lake Erie, Lake Ontario and Detroit River). Bacterial families with less than 0.1% relative abundance are combined and presented as “others”. **Fig S3.** LASSO regression analysis was used to indentify the best predictor variables for gut samples based on their alpha (Shannon entropy, PD, Chao1) and beta diversity (PCoA 1- 5) indices. Inside each bar is showing the coeffitient value and X axis is showing the importance of the predictor variable for alpha and beta diversity indices. Diet, location and fish species was identified as the best predictor for most of the diversity indecies. **Fig S4.** LASSO regression analysis was used to indentify the best predictor variables for fish skin samples based on their alpha (Shannon entropy, PD, Chao1) and beta diversity (PCoA 1- 5) indices. Inside each bar is showing the coeffitient value and X axis is showing the importance of the predictor variable for alpha and beta diversity indices. Location and fish species was identified as the best predictor for most of the diversity indecies. **Figure S5.** Scatterplot of pairwise host phylogenetic distance vs pairwise Bray Curtis dissimilarity for both gut (a) and skin (b) samples. Samples were combined within host species. Host phylogenetic distance was estimated using of CO1 and CytB mitochondrial gene sequences. **Table S1.** Summary of Great Lakes fish species sampled for gut and skin microbiome. We provide a description of the sample locations and total sample size. **Table S2.** Comparison of differentially abundant bacterial taxa at the family level for gut and skin microbiomes across all fish species and sample locations using DESeq2 method (Benjamini-Hochberg false-discovery rate [BH FDR] 0.05, |log_2_fold change| > 2). Positive log_2_ FC indicate higher abundance in skin samples and negative log_2_ FC specify higher abundance in gut samples.

## Data Availability

The raw 16S rRNA gene sequencing data are available at the Sequence Read Archive of NCBI with PRJNA701818 BioProject accession number. For a complete list of packages and code for microbiome analyses, see https://github.com/javad30/Host-Species-and-Habitat-Shape-Fish--associated-Bacterial-Communities.
