# Supplementary material for: Host species and habitat shape fish-associated bacterial communities: phylosymbiosis between fish and their microbiome
Source: Microbiome. 2023 Nov 20;11:258. doi: 10.1186/s40168-023-01697-6 (PMC10658978; doi:10.1186/s40168-023-01697-6)
Supplement: Supplementary file 2 — Additional file 1: Figure S1. Relative abundance of bacterial community composition presented at the phylum level for gut, skin, and water microbiota (samples are combined across sample types). Phyla with less than 0.01% of relative abundance are combined and presented as “others”). Figure S2. Bacterial community composition (relative abundance at the family level) for gut, skin, and water microbiomes across all fish species collected at three sites in the Great Lakes (Lake Erie, Lake Ontario and Detroit River). Bacterial families with less than 0.1% relative abundance are combined and presented as “others”. Fig S3. LASSO regression analysis was used to indentify the best predictor variables for gut samples based on their alpha (Shannon entropy, PD, Chao1) and beta diversity (PCoA 1- 5) indices. Inside each bar is showing the coeffitient value and X axis is showing the importance of the predictor variable for alpha and beta diversity indices. Diet, location and fish species was identified as the best predictor for most of the diversity indecies. Fig S4. LASSO regression analysis was used to indentify the best predictor variables for fish skin samples based on their alpha (Shannon entropy, PD, Chao1) and beta diversity (PCoA 1- 5) indices. Inside each bar is showing the coeffitient value and X axis is showing the importance of the predictor variable for alpha and beta diversity indices. Location and fish species was identified as the best predictor for most of the diversity indecies. Figure S5. Scatterplot of pairwise host phylogenetic distance vs pairwise Bray Curtis dissimilarity for both gut (a) and skin (b) samples. Samples were combined within host species. Host phylogenetic distance was estimated using of CO1 and CytB mitochondrial gene sequences. Table S1. Summary of Great Lakes fish species sampled for gut and skin microbiome. We provide a description of the sample locations and total sample size. Table S2. Comparison of differentially abundant bacterial taxa [file 40168_2023_1697_MOESM1_ESM.docx]

**Phylosymbiosis between Microbial Communities and Fish; Spatial and Host Species Shape Bacterial Community Composition**

Javad Sadeghi^1^, Subba Rao Chaganti^2^, Timothy B. Johnson^3^, Daniel D Heath^1^,^4*^

1 Great Lakes Institute for Environmental Research, University of Windsor, Windsor, Canada

2 Cooperative Institute for Great Lakes Research, University of Michigan, Ann Arbor, MI, USA

3 Ontario Ministry of Natural Resources and Forestry, Glenora Fisheries Station, Picton, Ontario, Canada

4 Department of Integrative Biology, University of Windsor, Windsor, ON, Canada

Corresponding author: D. D. Heath, Great Lakes Institute for Environmental Research, University of

Windsor, Ontario N9B 3P4, Canada

e-mail: dheath@uwindsor.ca


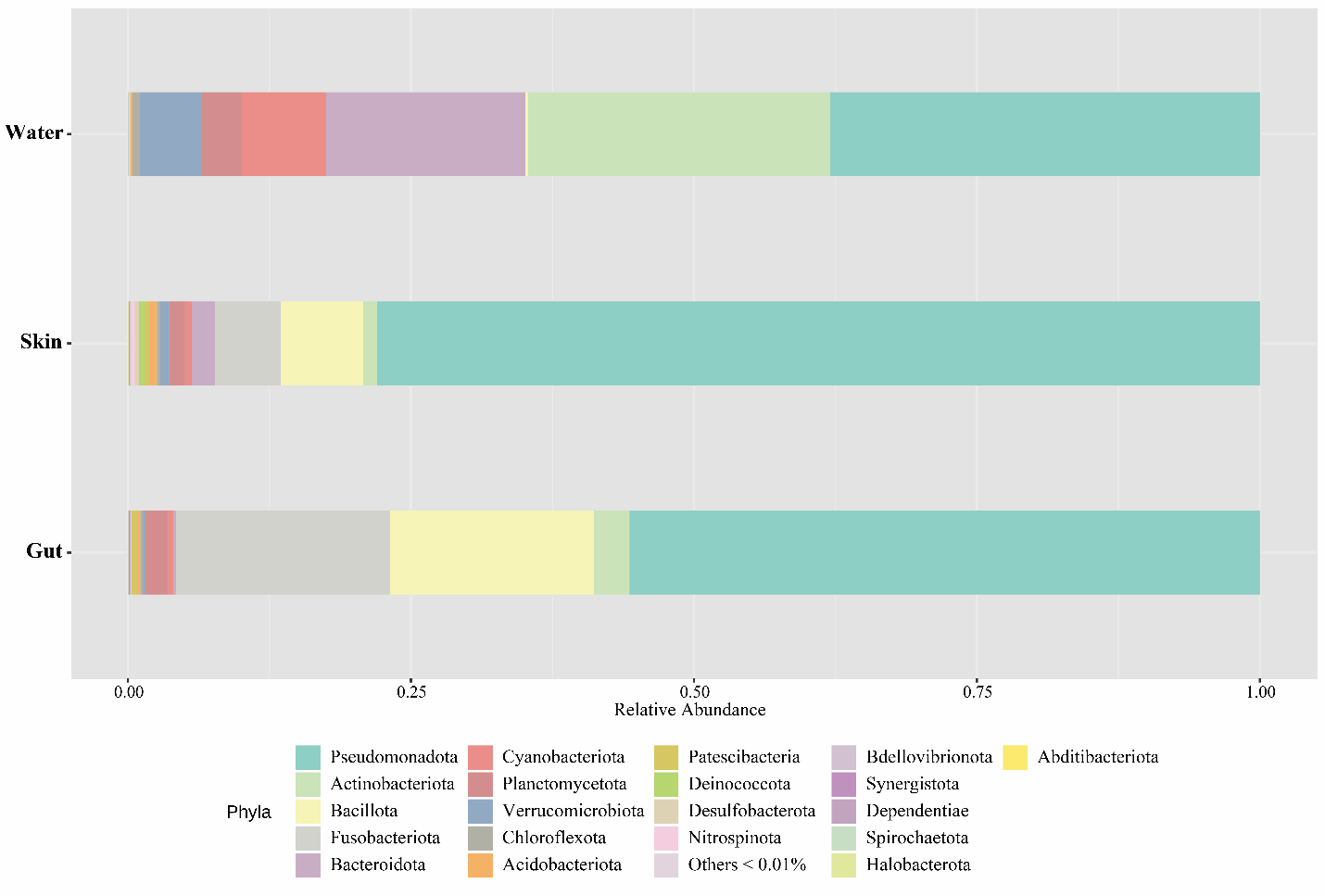


Figure S1: Relative abundance of bacterial community composition presented at the phylum level for gut, skin, and water microbiota (samples are combined across sample types). Phyla with less than 0.01% of relative abundance are combined and presented as “others”)


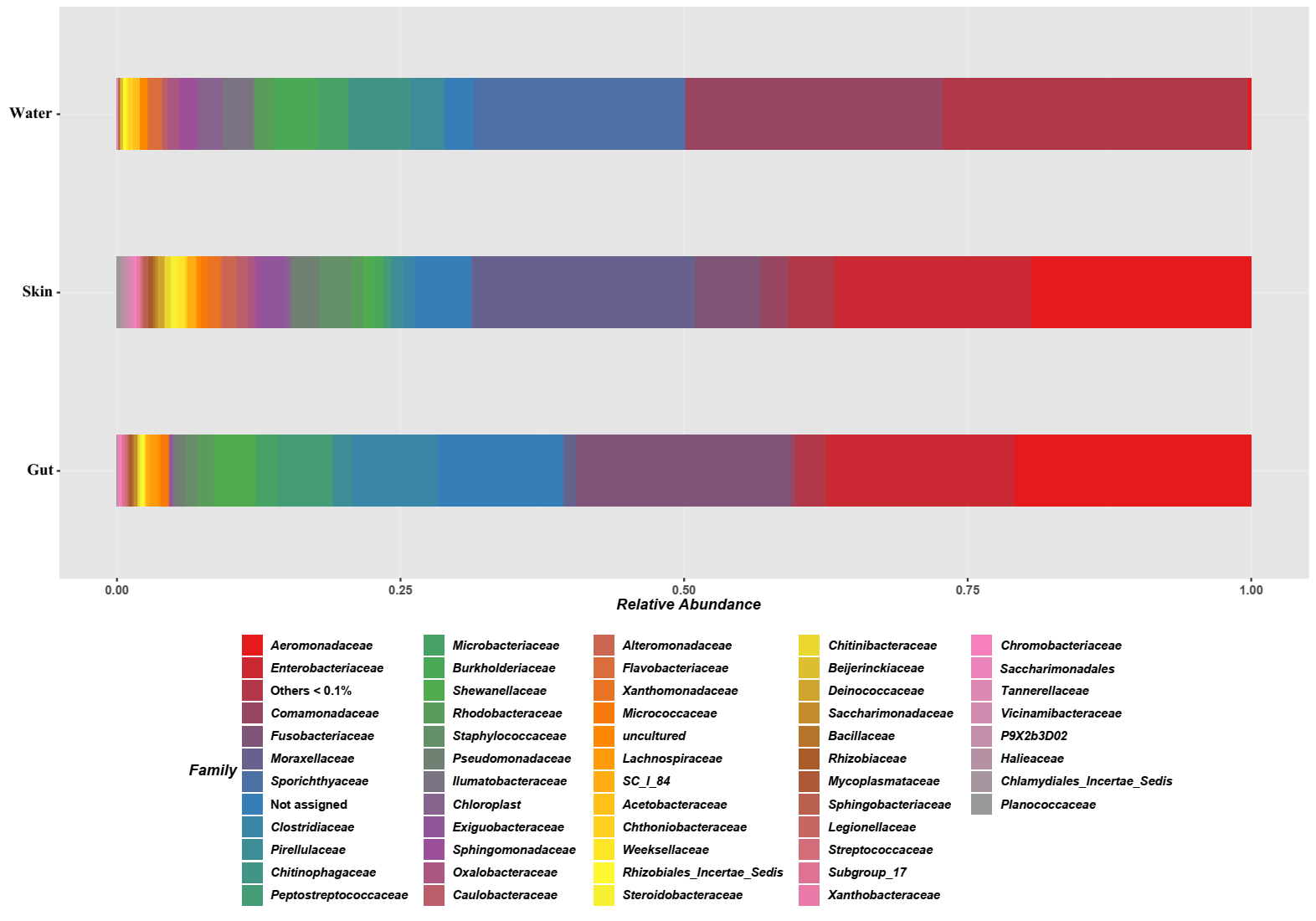


Figure S2: Bacterial community composition (relative abundance at the family level) for gut, skin, and water microbiomes across all fish species collected at three sites in the Great Lakes (Lake Erie, Lake Ontario and Detroit River). Bacterial families with less than 0.1% relative abundance are combined and presented as “others”.


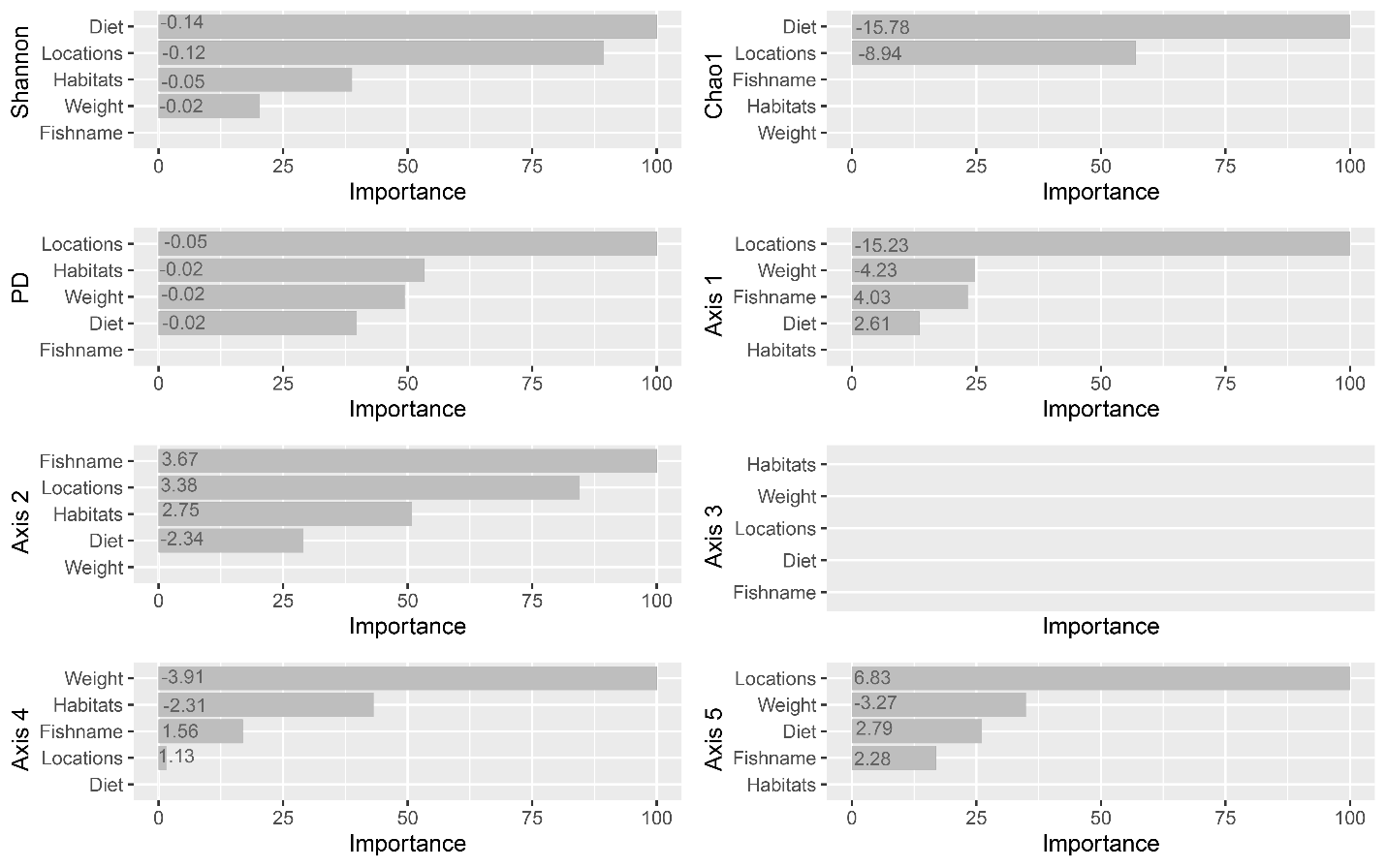


Fig S3. LASSO regression analysis was used to indentify the best predictor variables for gut samples based on their alpha (Shannon entropy, PD, Chao1) and beta diversity (PCoA 1- 5) indices. Inside each bar is showing the coeffitient value and X axis is showing the importance of the predictor variable for alpha and beta diversity indices. Diet, location and fish species was identified as the best predictor for most of the diversity indecies.


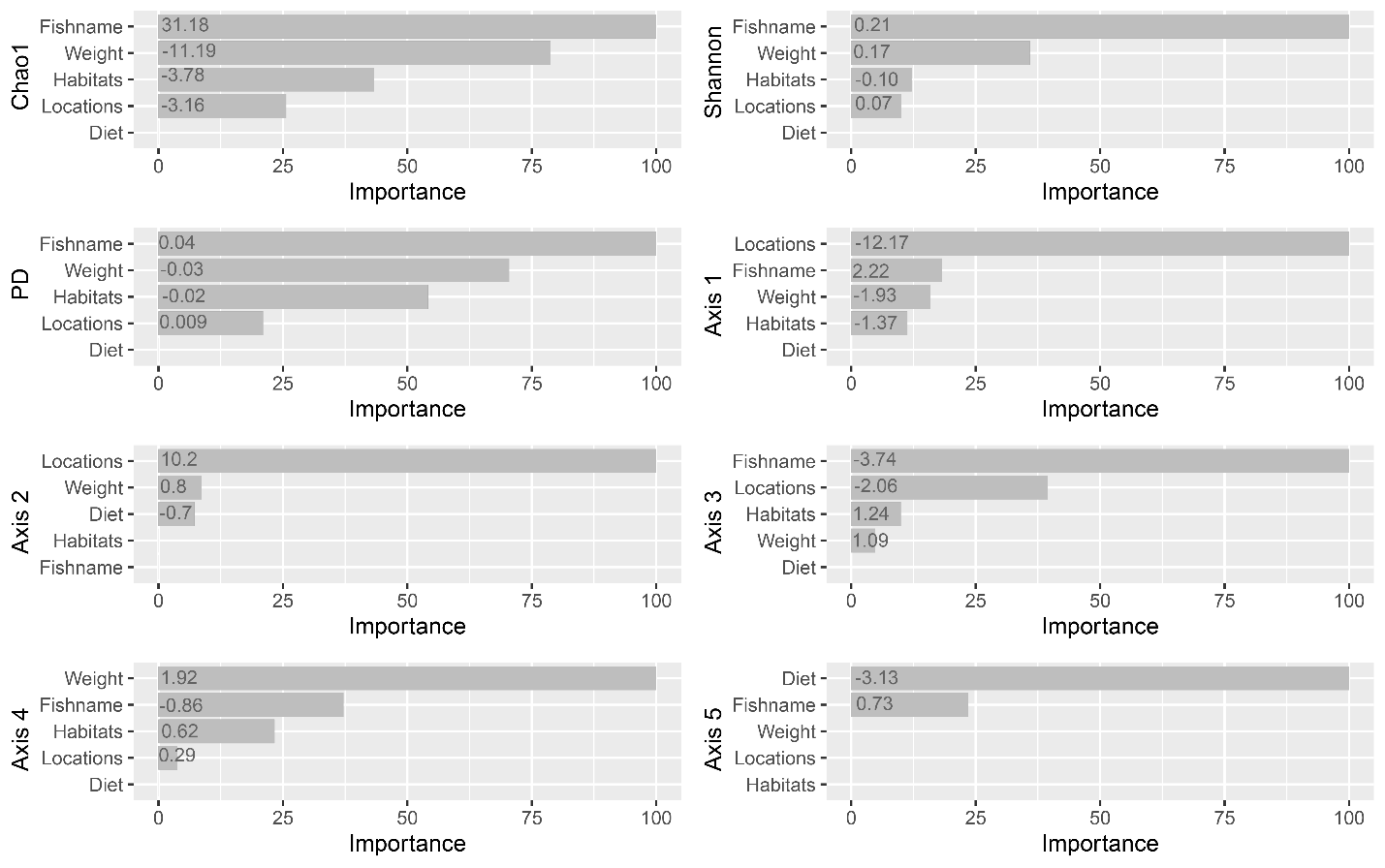


Fig S4. LASSO regression analysis was used to indentify the best predictor variables for fish skin samples based on their alpha (Shannon entropy, PD, Chao1) and beta diversity (PCoA 1- 5) indices. Inside each bar is showing the coeffitient value and X axis is showing the importance of the predictor variable for alpha and beta diversity indices. Location and fish species was identified as the best predictor for most of the diversity indecies.


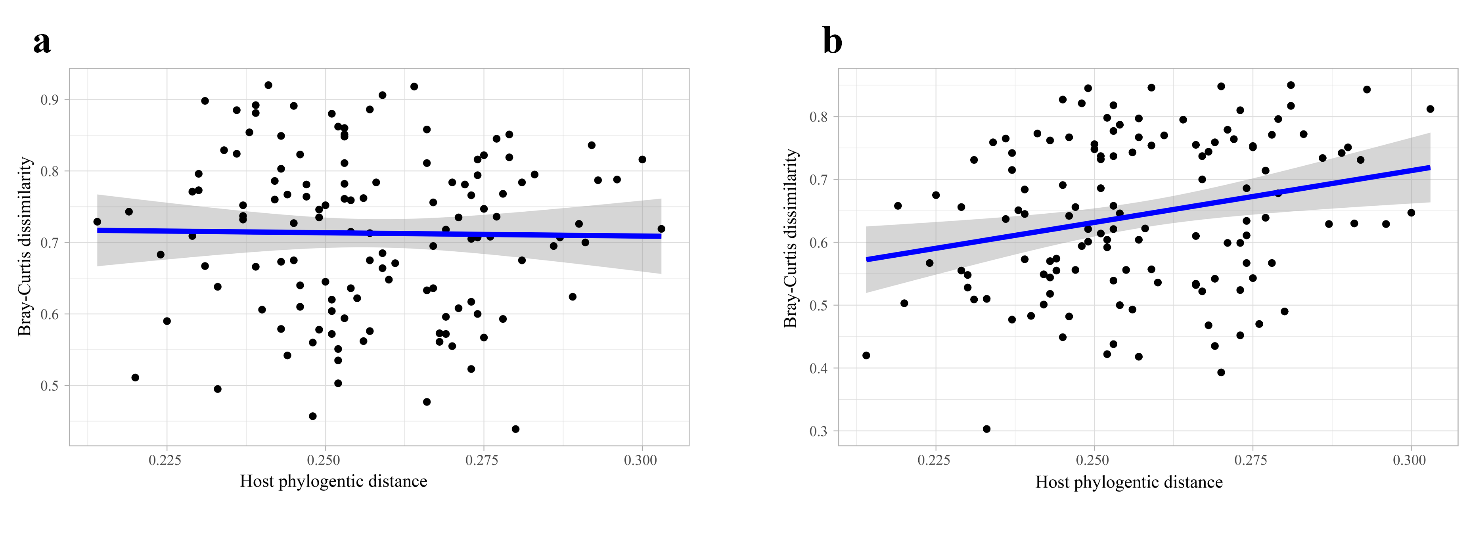


Figure S5. Scatterplot of pairwise host phylogenetic distance vs pairwise Bray Curtis dissimilarity for both gut (a) and skin (b) samples. Samples were combined within host species. Host phylogenetic distance was estimated using of CO1 and CytB mitochondrial gene sequences.

Table S1: Summary of Great Lakes fish species sampled for gut and skin microbiome. We provide a description of the sample locations and total sample size.

| Species | Locations | | | Number of samples |
| --- | --- | --- | --- | --- |
|  | Detroit River | Lake Erie | Lake Ontario |  |
| Alewife (*Alosa pseudoharengus*) | 0 | 0 | 22 | 22 |
| Blacknose shiner (*Notropis* *heterolepis*) | 11 | 0 | 0 | 11 |
| Brook silver (*Labidesthes sicculus*) | 7 | 0 | 0 | 7 |
| Brown trout (*Salmo* *trutta*) | 0 | 0 | 16 | 16 |
| Emerald shiner (*Notropis* *atherinoides*) | 15 | 0 | 0 | 15 |
| Freshwater Drum (*Aplodinotus* *grunniens*) | 5 | 17 | 10 | 32 |
| Gizzard Shad (*Dorosoma* *cepedianum*) | 10 | 0 | 2 | 12 |
| Lake trout (*Salvelinus* *namaycush*) | 0 | 0 | 24 | 24 |
| Pumpkinseed (*Lepomis* *gibbosus*) | 11 | 0 | 5 | 16 |
| Rock Bass (*Ambloplites* *rupestris*) | 7 | 0 | 3 | 10 |
| Round goby (*Neogobius* *melanostomus*) | 0 | 8 | 16 | 24 |
| Spotfin shiner (*Cyprinella* *spiloptera*) | 12 | 0 | 0 | 12 |
| Walleye (*Sander* *vitreus*) | 0 | 19 | 7 | 26 |
| White Bass (*Morone* *chrysops*) | 0 | 10 | 11 | 21 |
| White perch (*Morone* *americana*) | 0 | 21 | 12 | 33 |
| White sucker (*Catostomus* *commersonii*) | 10 | 0 | 2 | 12 |
| Yellow perch (*Perca* *flavescens*) | 10 | 15 | 16 | 41 |
| Total | 98 | 90 | 146 | 334 |

Table S2: Comparison of differentially abundant bacterial taxa at the family level for gut and skin microbiomes across all fish species and sample locations using DESeq2 method (Benjamini-Hochberg false-discovery rate [BH FDR] 0.05, |log_2_fold change| > 2). Positive log_2_ FC indicate higher abundance in skin samples and negative log_2_ FC specify higher abundance in gut samples.

| Family | log_2_ FC | padj |
| --- | --- | --- |
| *Deinococcaceae* | 5.7 | 2.00E-169 |
| *Exiguobacteraceae* | 5.6 | 4.00E-144 |
| *Alteromonadaceae* | 5 | 2.00E-98 |
| *Moraxellaceae* | 4.9 | 2.00E-86 |
| *Oxalobacteraceae* | 4.6 | 1.00E-135 |
| *Caulobacteraceae* | 4 | 2.00E-84 |
| *Weeksellaceae* | 3.3 | 2.00E-50 |
| *Sphingobacteriaceae* | 3.2 | 3.00E-68 |
| *Xanthomonadaceae* | 2.7 | 4.00E-49 |
| *Rickettsiaceae* | 2.7 | 1.00E-44 |
| *Flavobacteriaceae* | 2.5 | 1.00E-30 |
| *Alcaligenaceae* | 2.4 | 1.00E-40 |
| *Devosiaceae* | 2.3 | 6.00E-41 |
| *Pseudomonadaceae* | 2.1 | 9.00E-28 |
| *Xanthobacteraceae* | 2.1 | 7.00E-25 |
| *Comamonadaceae* | 2 | 3.00E-36 |
| *P9X2b3D02* (Nitrospinota) | 2 | 8.00E-35 |
| *Microbacteriaceae* | -5.7 | 9.00E-139 |
| *Lachnospiraceae* | -4.3 | 1.00E-72 |
| *PeM15* (Actinobacteria) | -4.3 | 7.00E-118 |
| *Rhizobiales* | -4.1 | 1.00E-106 |
| *Cyanobiaceae* | -3.4 | 2.00E-69 |
| *Clostridiaceae* | -3.3 | 2.00E-40 |
| *Peptostreptococcaceae* | -3.3 | 2.00E-36 |
| *Subgroup_17* (Vicinamibacteria) | -3.1 | 9.00E-65 |
| *Pirellulaceae* | -3.1 | 6.00E-50 |
| *Caldilineaceae* | -3 | 9.00E-68 |
| *Microcystaceae* | -3 | 4.00E-53 |
| *Saccharimonadales* | -2.9 | 6.00E-56 |
| *Mycoplasmataceae* | -2.8 | 1.00E-38 |
| *Isosphaeraceae* | -2.6 | 1.00E-46 |
| *IMCC26256* (Acidimicrobiia) | -2.6 | 7.00E-58 |
| *Saccharimonadaceae* | -2.6 | 5.00E-42 |
| *1_20* (Anaerolineae) | -2.4 | 1.00E-40 |
| *Gemmataceae* | -2.3 | 3.00E-39 |
| *Holosporaceae* | -2 | 5.00E-33 |
| *Fusobacteriaceae* | -2 | 6.00E-15 |
